# Correction: A Robust Model-free Approach for Rare Variants Association Studies Incorporating Gene-Gene and Gene-Environmental Interactions

**DOI:** 10.1371/journal.pone.0098083

**Published:** 2014-05-14

**Authors:** The PLOS ONE Staff

The link to the R code of the proposed test scores in the final sentence of the Discussion section is incorrect. The correct link to the download is: http://www.columbia.edu/~rf2283/Software.html
