# Supplementary material for: A Robust Model-free Approach for Rare Variants Association Studies Incorporating Gene-Gene and Gene-Environmental Interactions
Source: PLoS One. 2013 Dec 17;8(12):e83057. doi: 10.1371/journal.pone.0083057 (PMC3866272; doi:10.1371/journal.pone.0083057)

Supporting Information

A Robust Model-free Approach for Rare Variants Association Studies Incorporating Gene-Gene and Gene-Environmental Interactions

Ruixue Fan and Shaw-Hwa Lo*

Department of Statistics, Columbia University, New York, NY 10027, USA
*Corresponding author.

Email addresses:
RF: rf2283@columbia.edu
SHL: slo@stat.columbia.edu

Supplementary Tables:

Table S1. Models to generate simulated phenotypes

Null Models		
	Dichotomous	Continuous
Null-1 (null setting for I_1, I_2, $p*$ and I_2^*-*Global*)	$\log\left(\frac{P(A)}{1-P(A)}\right) = \log(1/99)$	$y = 0.5*U_1 + 0.5*U_2 + \varepsilon$
Null-2 (null setting for I_2^*-*Local*)	$\log\left(\frac{P(A)}{1-P(A)}\right) = \log(1/99) + \log(2)*E$	N/A
Genetic Marginal Effect Models		
	Dichotomous	Continuous
Scenario 1 (constant marginal effects)	$\log\left(\frac{P(A)}{1-P(A)}\right) = \log(1/99) + \log(3)*\sum_{i=1}^{5} X_i$	$y = 0.5*U_1 + 0.5*U_2 + 0.8*\sum_{i=1}^{5} X_i + \varepsilon$
Scenario 2 (marginal effects negatively correlated with MAF)	$\log\left(\frac{P(A)}{1-P(A)}\right) = \log(1/99) + \sum_{i=1}^{5} \beta_i X_i$ where $\beta_i = \lvert\log_{10} MAF\rvert * \ln 5/4$	$y = 0.5*U_1 + 0.5*U_2 + \sum_{i=1}^{5} \beta_i X_i + \varepsilon$, where $\beta_i = \lvert\log_{10} MAF\rvert * 0.4$
Scenario 3 (both positive and negative marginal effect)	$\log\left(\frac{P(A)}{1-P(A)}\right) = \log(1/99) + \sum_{i=1}^{5} \beta_i X_i - \sum_{i=6}^{10} \beta_i X_i$ where $\beta_i = \lvert\log_{10} MAF\rvert * \ln 5/4$	$y = 0.5*U_1 + 0.5*U_2 + \sum_{i=1}^{5} \beta_i X_i - \sum_{i=6}^{10} \beta_i X_i + \varepsilon$, where $\beta_i = \lvert\log_{10} MAF\rvert * 0.4$

G×G Interaction Effect Models		
	Dichotomous	Continuous
Scenario 4 (50% SNPs participate in G×G interaction effects)	$\log\left(\frac{P(A)}{1-P(A)}\right)=\log(1/99)+\sum_{i=1}^{5}\sum_{j=6}^{10}\log 5*I(X_i>0,X_j>0)*(-1)^i,$	$y=0.5*U_1+0.5*U_2+\sum_{i=1}^{5}\sum_{j=6}^{10}1.5*I(X_i>0,X_j>0)*(-1)^i+\varepsilon$
Scenario 5 (75% SNPs participate in G×G interaction effects)	$\log\left(\frac{P(A)}{1-P(A)}\right)=\log(1/99)+\sum_{i=1}^{6}\sum_{j=7}^{15}\log 5*I(X_i>0,X_j>0)*(-1)^i,$	$y=0.5*U_1+0.5*U_2+\sum_{i=1}^{6}\sum_{j=7}^{15}1.5*I(X_i>0,X_j>0)*(-1)^i+\varepsilon$
Scenario 6 (Both marginal and G×G interaction effects)	$\log\left(\frac{P(A)}{1-P(A)}\right)=\log\frac{1}{99}+\left(\sum_{i=1}^{2}\beta_iX_i-\sum_{i=3}^{5}\beta_iX_i\right)*0.1+\sum_{i=1}^{6}\sum_{j=7}^{15}\log 5*I(X_i>0,X_j>0)*(-1)^i$ where $\beta_i=\lvert\log_{10}MAF\rvert*\ln 5/4$	$y=0.5*U_1+0.5*\mathrm{U}_2+\left(\sum_{i=1}^{2}\beta_iX_i-\sum_{i=3}^{5}\beta_iX_i\right)*0.1+\sum_{i=1}^{6}\sum_{j=7}^{15}1.5*I(X_i>0,X_j>0)*(-1)^i+\varepsilon$ where $\beta_i=\lvert\log_{10}MAF\rvert*0.4$
G×E Interaction Effect Models		
	Dichotomous	Continuous
Scenario 7 (Positive G×E effect)	$\log\left(\frac{P(A)}{1-P(A)}\right)=\log(1/99)+\log(2)*E+\sum_{i=1}^{5}\beta_i*E*X_i$ where $\beta_i=\lvert\log_{10}MAF\rvert*\ln 5/4$	N/A
Scenario 8 (Positive and negative G×E effect)	$\log\left(\frac{P(A)}{1-P(A)}\right)=\log(1/99)+\log 2*E+\sum_{i=1}^{5}\beta_i*E*X_i-\sum_{i=6}^{10}\beta_i*E*X_i$ where $\beta_i=\lvert\log_{10}MAF\rvert*\ln 5/4$	N/A

We generated 20 independent SNPs with MAF uniformly distributed in 0.0001 and 0.01 except in scenarios 4 and 5 where all the MAFs are set as 0.01. For models involving environmental factors, the environmental factor E is generated from a Bernoulli distribution with success probability 0.5. In models for continuous traits, $U_1 \sim N(0,1)$ and $U_2 \sim Bernoulli(0.5)$ are two covariates independent of genetic factors and $\varepsilon \sim N(0,1)$ is the random noise.

Table S2. Power of different methods for dichotomous traits in scenarios 1~6 (α=0.05)

Scenario	Sample Size	CMC	WS	VT	RB	SKAT	SKATint	I_1	I_2	p^*
1	600	0.709	0.775	0.584	0.699	0.774	0.690	0.813	0.666	0.786
1	1000	0.822	0.868	0.724	0.838	0.918	0.884	0.934	0.832	0.925
1	1500	0.924	0.945	0.855	0.948	0.974	0.967	0.976	0.941	0.973
1	2000	0.946	0.963	0.92	0.967	0.981	0.980	0.979	0.956	0.981
2	600	0.474	0.533	0.366	0.442	0.504	0.449	0.537	0.402	0.512
2	1000	0.683	0.740	0.545	0.694	0.744	0.688	0.768	0.621	0.742
2	1500	0.773	0.807	0.680	0.791	0.862	0.832	0.856	0.718	0.834
2	2000	0.889	0.916	0.813	0.913	0.957	0.950	0.956	0.874	0.948
3	600	0.247	0.298	0.152	0.324	0.616	0.590	0.616	0.559	0.616
3	1000	0.370	0.422	0.232	0.534	0.881	0.849	0.880	0.794	0.867
3	1500	0.438	0.491	0.277	0.779	0.980	0.973	0.975	0.946	0.976
3	2000	0.589	0.644	0.399	0.924	0.998	0.999	0.999	0.988	0.999
4	600	0.063	0.088	0.077	0.088	0.079	0.094	0.083	0.087	0.088
4	1000	0.080	0.108	0.099	0.094	0.099	0.112	0.105	0.128	0.124
4	1500	0.087	0.123	0.101	0.133	0.147	0.165	0.157	0.238	0.207
4	2000	0.084	0.122	0.112	0.143	0.156	0.200	0.168	0.311	0.271
5	600	0.152	0.265	0.301	0.351	0.317	0.337	0.351	0.344	0.342
5	1000	0.211	0.364	0.464	0.557	0.503	0.563	0.537	0.683	0.633
5	1500	0.287	0.505	0.633	0.755	0.709	0.791	0.744	0.918	0.899
5	2000	0.361	0.607	0.752	0.884	0.845	0.909	0.874	0.972	0.968
6	600	0.165	0.285	0.314	0.361	0.319	0.352	0.347	0.363	0.360
6	1000	0.222	0.372	0.455	0.567	0.493	0.579	0.536	0.689	0.646
6	1500	0.278	0.503	0.628	0.771	0.740	0.803	0.784	0.929	0.890
6	2000	0.349	0.567	0.726	0.886	0.871	0.923	0.890	0.976	0.972

Table S3. Power of different methods for dichotomous traits in scenarios 1~6 (α=0.01)

Scenario	Sample Size	CMC	WS	VT	RB	SKAT	SKATint	I_1	I_2	p^*
1	600	0.470	0.541	0.364	0.494	0.604	0.492	0.664	0.452	0.629
1	1000	0.651	0.699	0.556	0.677	0.812	0.743	0.832	0.662	0.817
1	1500	0.804	0.838	0.732	0.865	0.937	0.914	0.942	0.846	0.938
1	2000	0.869	0.898	0.802	0.928	0.960	0.954	0.961	0.894	0.957
2	600	0.253	0.298	0.182	0.239	0.275	0.221	0.312	0.204	0.282
2	1000	0.436	0.494	0.306	0.464	0.546	0.468	0.592	0.388	0.536
2	1500	0.553	0.604	0.458	0.609	0.703	0.665	0.704	0.510	0.671
2	2000	0.730	0.767	0.649	0.801	0.879	0.866	0.880	0.695	0.867
3	600	0.101	0.134	0.049	0.139	0.376	0.349	0.383	0.327	0.366
3	1000	0.170	0.207	0.091	0.291	0.711	0.669	0.717	0.607	0.700
3	1500	0.224	0.270	0.139	0.502	0.918	0.906	0.915	0.854	0.912
3	2000	0.353	0.400	0.233	0.767	0.986	0.987	0.986	0.960	0.986
4	600	0.016	0.023	0.022	0.027	0.030	0.037	0.025	0.034	0.029
4	1000	0.016	0.027	0.019	0.022	0.020	0.026	0.018	0.029	0.024
4	1500	0.014	0.030	0.033	0.038	0.049	0.061	0.054	0.089	0.074
4	2000	0.025	0.032	0.024	0.041	0.054	0.066	0.055	0.120	0.096
5	600	0.047	0.099	0.138	0.172	0.141	0.160	0.153	0.169	0.172
5	1000	0.062	0.159	0.255	0.320	0.260	0.323	0.290	0.434	0.400
5	1500	0.105	0.248	0.404	0.534	0.504	0.589	0.561	0.781	0.752
5	2000	0.143	0.352	0.545	0.712	0.678	0.769	0.722	0.934	0.910
6	600	0.041	0.099	0.131	0.180	0.137	0.146	0.160	0.153	0.157

6	1000	0.069	0.168	0.240	0.341	0.273	0.321	0.304	0.446	0.401
6	1500	0.090	0.244	0.385	0.555	0.513	0.611	0.563	0.789	0.757
6	2000	0.150	0.340	0.531	0.705	0.687	0.793	0.732	0.938	0.917

Table S4. Power of different methods for continuous traits in scenarios 1~6 (α=0.05)

Scenario	Sample Size	CMC	WS	VT	SKAT	SKATint	I_1	I_2	p^*
1	600	0.409	0.568	0.523	0.604	0.584	0.583	0.445	0.544
1	1000	0.608	0.744	0.716	0.838	0.824	0.817	0.642	0.790
1	1500	0.763	0.873	0.874	0.945	0.949	0.927	0.752	0.913
1	2000	0.833	0.933	0.939	0.970	0.971	0.956	0.778	0.952
2	600	0.458	0.626	0.597	0.710	0.682	0.694	0.593	0.679
2	1000	0.668	0.839	0.831	0.892	0.891	0.874	0.774	0.851
2	1500	0.815	0.926	0.922	0.977	0.976	0.970	0.892	0.958
2	2000	0.919	0.971	0.977	0.989	0.990	0.982	0.932	0.981
3	600	0.086	0.071	0.215	0.969	0.965	0.957	0.915	0.957
3	1000	0.113	0.080	0.321	0.996	0.997	0.996	0.989	0.997
3	1500	0.162	0.089	0.429	1.000	1.000	1.000	0.998	1.000
3	2000	0.160	0.086	0.537	1.000	1.000	1.000	1.000	1.000
4	600	0.045	0.041	0.050	0.118	0.193	0.110	0.296	0.252
4	1000	0.052	0.044	0.048	0.142	0.250	0.144	0.449	0.392
4	1500	0.059	0.045	0.061	0.178	0.294	0.174	0.579	0.508
4	2000	0.045	0.034	0.056	0.200	0.363	0.206	0.695	0.629
5	600	0.051	0.085	0.109	0.328	0.543	0.331	0.664	0.637
5	1000	0.039	0.063	0.116	0.380	0.619	0.370	0.861	0.810
5	1500	0.051	0.090	0.119	0.504	0.742	0.509	0.952	0.926
5	2000	0.045	0.081	0.150	0.631	0.844	0.627	0.987	0.980
6	600	0.046	0.078	0.090	0.319	0.508	0.312	0.675	0.641
6	1000	0.057	0.071	0.106	0.440	0.653	0.432	0.863	0.826
6	1500	0.037	0.064	0.115	0.539	0.769	0.523	0.949	0.931
6	2000	0.047	0.077	0.144	0.701	0.879	0.700	0.991	0.982

Table S5. Power of different methods for continuous traits in scenarios 1~6 (α=0.01)

Scenario	Sample Size	CMC	WS	VT	SKAT	SKATint	I_1	I_2	p^*
1	600	0.211	0.334	0.294	0.377	0.344	0.362	0.206	0.334
1	1000	0.403	0.514	0.492	0.680	0.655	0.663	0.401	0.631
1	1500	0.559	0.688	0.697	0.859	0.864	0.832	0.538	0.813
1	2000	0.673	0.803	0.823	0.929	0.932	0.912	0.552	0.891
2	600	0.242	0.362	0.346	0.485	0.454	0.459	0.343	0.443
2	1000	0.443	0.629	0.632	0.743	0.750	0.711	0.528	0.692
2	1500	0.630	0.798	0.814	0.927	0.939	0.908	0.671	0.894
2	2000	0.802	0.907	0.924	0.971	0.978	0.964	0.789	0.961
3	600	0.028	0.012	0.081	0.892	0.869	0.864	0.776	0.860
3	1000	0.035	0.028	0.172	0.993	0.989	0.990	0.948	0.985
3	1500	0.054	0.024	0.249	1.000	1.000	0.999	0.989	0.999
3	2000	0.069	0.026	0.361	1.000	1.000	1.000	0.996	1.000
4	600	0.006	0.008	0.015	0.045	0.083	0.041	0.157	0.124
4	1000	0.009	0.007	0.010	0.047	0.101	0.047	0.262	0.208
4	1500	0.012	0.009	0.012	0.057	0.135	0.063	0.348	0.299
4	2000	0.008	0.004	0.013	0.088	0.175	0.084	0.477	0.413

5	600	0.008	0.030	0.029	0.147	0.321	0.138	0.509	0.453
5	1000	0.009	0.019	0.027	0.192	0.409	0.190	0.694	0.646
5	1500	0.012	0.018	0.035	0.278	0.518	0.279	0.854	0.825
5	2000	0.015	0.026	0.048	0.430	0.676	0.420	0.947	0.928
6	600	0.011	0.018	0.030	0.151	0.299	0.148	0.484	0.420
6	1000	0.007	0.021	0.030	0.243	0.452	0.246	0.740	0.679
6	1500	0.009	0.020	0.040	0.335	0.571	0.326	0.866	0.841
6	2000	0.007	0.027	0.058	0.470	0.740	0.467	0.957	0.948

Table S6. Power of different methods for dichotomous traits in G×E interaction effect models (α=0.05)

Scenario	Sample Size	CMC	WS	VT	RB	SKAT	SKATint	I_1	I_2	p*	I_2^*- Global	I_2^*- Local
7	600	0.299	0.337	0.209	0.239	0.218	0.220	0.219	0.178	0.207	0.543	0.362
7	1000	0.418	0.468	0.323	0.377	0.338	0.324	0.408	0.309	0.386	0.852	0.593
7	1500	0.551	0.601	0.439	0.547	0.438	0.434	0.580	0.425	0.550	0.967	0.770
7	2000	0.636	0.679	0.515	0.609	0.543	0.521	0.653	0.487	0.646	0.968	0.813
8	600	0.149	0.184	0.108	0.165	0.307	0.313	0.298	0.236	0.300	0.565	0.381
8	1000	0.214	0.246	0.152	0.262	0.471	0.481	0.483	0.396	0.483	0.837	0.640
8	1500	0.278	0.303	0.175	0.347	0.598	0.602	0.625	0.507	0.612	0.915	0.732
8	2000	0.323	0.360	0.194	0.478	0.767	0.764	0.794	0.678	0.780	0.980	0.872

Table S7. Power of different methods for dichotomous traits in G×E interaction effect models (α=0.01)

Scenario	Sample Size	CMC	WS	VT	RB	SKAT	SKATint	I_1	I_2	p*	I_2^*- Global	I_2^*- Local
7	600	0.100	0.125	0.07	0.074	0.083	0.084	0.076	0.053	0.096	0.29	0.154
7	1000	0.207	0.240	0.154	0.189	0.130	0.123	0.182	0.102	0.195	0.615	0.332
7	1500	0.304	0.351	0.220	0.326	0.242	0.221	0.344	0.201	0.374	0.870	0.524
7	2000	0.384	0.419	0.283	0.395	0.313	0.302	0.429	0.234	0.460	0.900	0.587
8	600	0.048	0.060	0.033	0.048	0.125	0.131	0.107	0.070	0.132	0.309	0.172
8	1000	0.081	0.097	0.054	0.104	0.251	0.253	0.234	0.176	0.288	0.643	0.361
8	1500	0.123	0.134	0.058	0.152	0.376	0.374	0.379	0.254	0.425	0.765	0.501
8	2000	0.126	0.146	0.072	0.222	0.548	0.557	0.556	0.412	0.616	0.940	0.676

Supplementary Figures

Figure S1. Type I error for different methods in various sample sizes with nominal α levels 0.05 (left) and 0.01(right). Results for four sample sizes: 600, 1000, 1500 and 2000, with equal numbers of cases and controls.

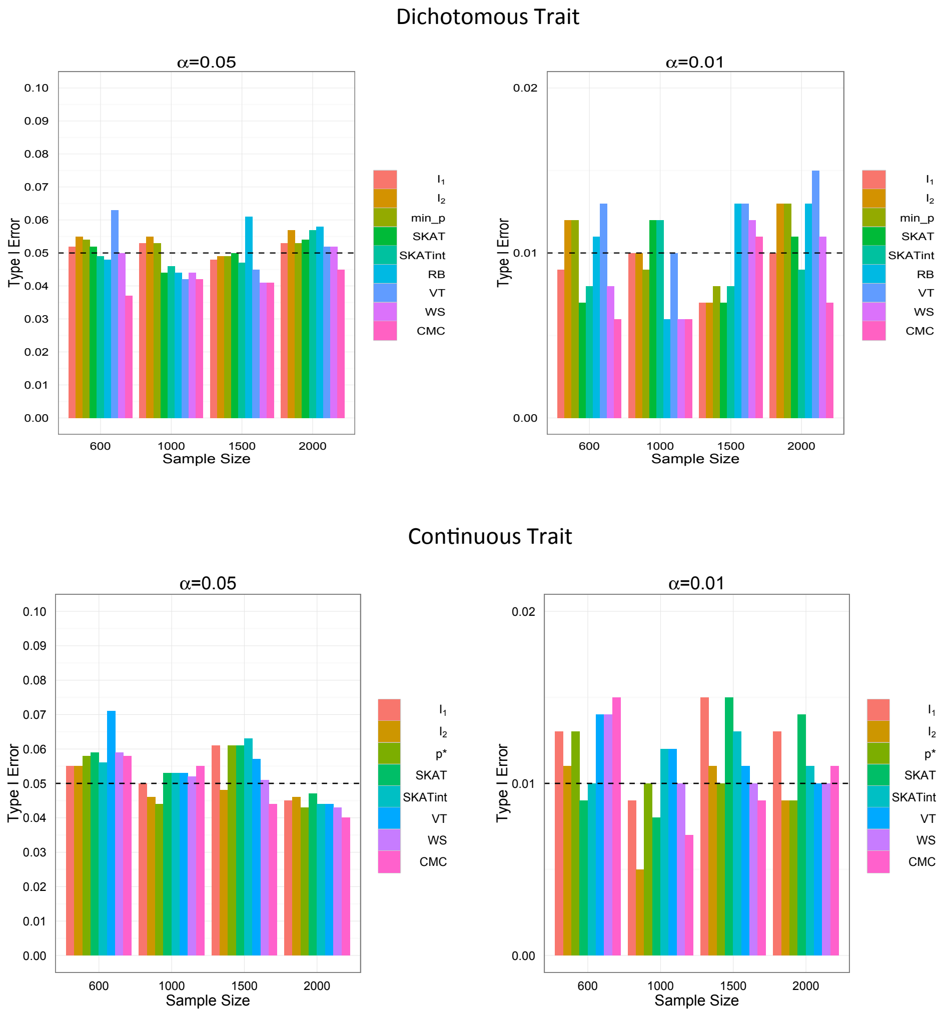

Figure S2. Power comparison in scenarios 1~6 for dichotomous traits with 500 cases and 500 controls when the SNP number is 30. Powers were calculated for nominal α levels 0.05 (left) and 0.01(right). Power was evaluated for I_1, I_2, p^*, SKAT, SKATint, VT, RB, WS and CMC. P-values were estimated using 10,000 permutations and power was evaluated using 1,000 replicates.

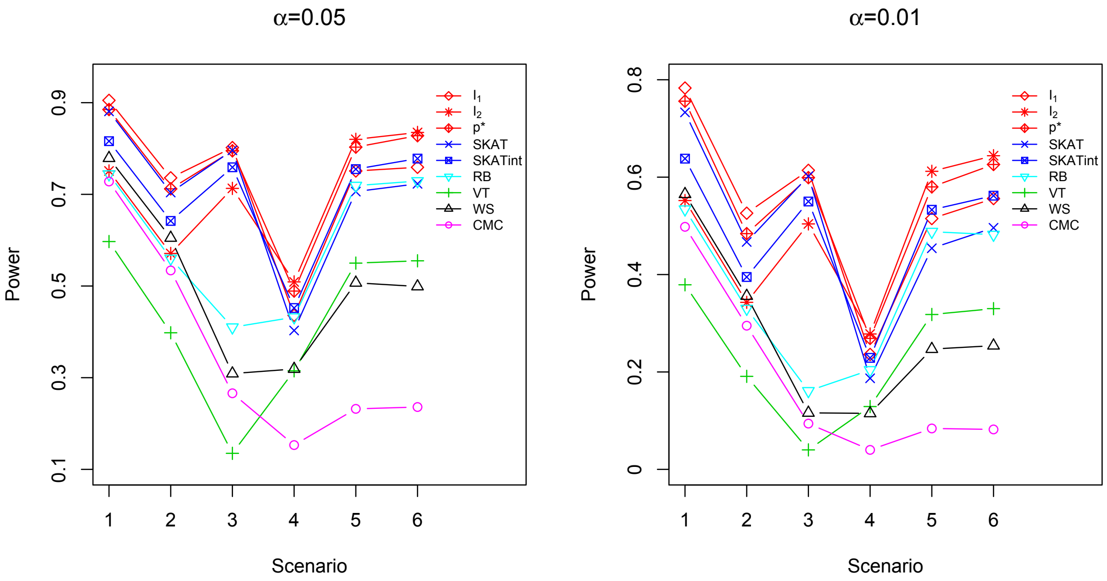

Supplement: File S1 — The supporting information file for article “A Robust Model-free Approach for Rare Variants Association Studies Incorporating Gene-Gene and Gene-Environmental Interactions”. It contains the files: Table S1. Models to generate simulated phenotypes; Table S2. Power of different methods for dichotomous traits in scenarios 1∼6 (α = 0.05); Table S3. Power of different methods for dichotomous traits in scenarios 1∼6 (α = 0.01); Table S4. Power of different methods for continuous traits in scenarios 1∼6 (α = 0.05); Table S5. Power of different methods for continuous traits in scenarios 1∼6 (α = 0.01); Table S6. Power of different methods for dichotomous traits in G×E interaction effect models (α = 0.05); Figure S1: Type I error for different methods in various sample sizes with nominal α levels 0.05 (left) and 0.01(right); Figure S2: Power comparison in scenarios 1∼6 for dichotomous traits with 500 cases and 500 controls when the SNP number is 30. (DOC) [file pone.0083057.s001.doc]
